# Female medical students' awareness, attitudes, and knowledge about early detection of breast cancer in Syrian Private University, Syria

**DOI:** 10.1016/j.heliyon.2020.e03819

**Published:** 2020-04-24

**Authors:** Abdullah Omar, Aliaa Bakr, Nazir Ibrahim

**Affiliations:** a Syrian Private University, Faculty of Medicine, Damascus, Syria; b Internal Medicine Department, Oncology Medicine, Damascus University, Damascus, Syria

**Keywords:** Health sciences, Public health, Women's health, Oncology, Clinical research, Medical students, Breast cancer, Acknowledge, Awareness, Syria, Heliyon

## Abstract

**Introduction:**

Breast cancer is the most frequent cancer among women, impacting 2.1 million women each year, and also causes the greatest number of cancer-related deaths among women. The study aims to determine the community awareness levels in our country and extrapolate knowledge and awareness about the methods of early detection.

**Methods:**

It was performed as cross-sectional study in Syrian Private University in Damascus, Syria from January to March 2019. The population included female students of all years in medical faculties: medicine, pharmacy and dentistry collages. Data collection have been done by breast cancer awareness measure (BCAM) questionnaire.

**Results:**

The total participants were 407 divided into three faculties. The average knowledge rate was (57.5%). (70 %) of participants were not familiar with mammography. It is a good result that (86.7 %) acquainted about self-examination and (94.8%) believe that it is very important. But it is obvious that the practical side is poor, there were (32.7%) of students who apply BSE.

**Conclusion:**

This study showed a lack of awareness among medical students. Perhaps the most important reason is the lack of awareness programs that must include all strata of society, especially students of medical colleges and doctors for their important role in spreading awareness to avoid this danger that surrounds our ladies.

## 1 Introduction

Breast cancer is the most frequent cancer among women, affecting 2.1 million women each year, and causes the greatest number of cancer-related deaths among women. In 2018, it is estimated that 627,000 women died from breast cancer – that is approximately 15% of all cancer deaths among women. While breast cancer rates are higher among women in more developed regions, rates are increasing in nearly every region globally [1]. Breast cancer comprises ~16% of all cases of cancer in women [2].

Early diagnosis strategies focus on providing timely access to cancer treatment by reducing barriers to care and/or improving access to effective diagnosis services. The goal is to increase the proportion of breast cancers identified at an early stage, allowing more effective treatment to be used and reducing the risks of death from breast cancer. World Health Organization Package of essential no communicable (PEN) disease interventions for primary health care in low-resource settings has guidance on the approach to assessment and referral for women with suspected breast cancer in the primary care setting [1].

The incidence of breast cancer is increasing in the developing world due to increasing life expectancy, increase urbanization and adoption of western lifestyles [1].

Although some risk reduction might be achieved with the prevention, these strategies cannot eliminate the majority of breast cancers that develop in low- and middle-income countries where breast cancer is diagnosed in very late stages [1].

In third world countries, such as in Africa and the Middle East, a higher proportion of patients are diagnosed below the age of 40, reaching as high as 20% [3].

Maybe religion, culture, excess privacy, fear and shame, these reasons and others prevent women in the Arab world to check out their doctor when the matter is regarding to their breasts.

Many types of research were performed in the Arab world to raise awareness of breast cancer like Saudi Arabia [4], Tunisia [5] …. unfortunately, they showed a lack of knowledge. This lack is not only in the Arab world, but there is also in low-resource countries like Africa and South Asia. In 2014, in Pokhara valley, Nepal, it was found that the level of awareness of breast cancer, including knowledge of warning signs and BSE, is sub-optimal among Nepalese women [6].

Several studies found that regular breast cancer screening (BCS) interventions could facilitate early detection and reduce its morbidity and mortality. One systematic review in 2015 proved that the overall mortality rates of the UK and US have been improved because of awareness, improved medical technology and screening, but in case of India and Egypt, the condition is less positive because of lack of awareness [7].

So according to the WHO, there are limited resource settings with weak health systems where breast cancer incidence is relatively low and the majority of women are diagnosed in late stages have the option to implement early diagnosis programs based on awareness of early signs and symptoms and prompt referral to diagnosis and treatment [1].

That encouraged us to determine the community awareness levels in our country and extrapolate knowledge and awareness about the methods of early detection. It had focused on the students of medical colleges because of their important roles as consular for their surrounding environment.

## 2 Methods

### 2.1 Setting and sampling

A cross-sectional study was performed in Syrian Private University in Damascus, Syria.

The data was collected from January to March 2019.

The population included female students of all levels (from first year to final year) in medical faculties: medicine, pharmacy and dentistry collages.

The study size was calculated by the application online "Sample Size calculator".

The simple randomization was performed method on female students according to the random distribution with sampling decimal (2).

### 2.2 Tools of study

Data collection was done by a survey which is the international version questionnaire of the breast cancer awareness measure (BCAM) that has been validated in 2018 again [8].

This survey was used in many studies [6, 9]. It was translated into Arabic and used in many studies in Arab regions [4, 5].

Ethical approval was obtained from Syrian Private University's (SPU) ethical committee.

The Data collection form was includes 67 questions divided into five sections (general information, signs and symptoms, risk factor, breast self-examination, feelings and fears), addition to socio-demographic and descriptive characteristics (age, educational level, marital status, having a relative doctor, having an affected relative, religion, curriculums -only medicine faculty- and habits).

### 2.3 Data analysis

Data analysis was carried out in SPSS version 25 and P-values <0.05 were considered statistically significant.

Each question in the questionnaire could be answered “yes”, “no”, or “I don't know”. Correct answers scored one point, while incorrect answers and “I don't know” received a score of zero. The total score ranged from zero to 55; higher scores indicated greater knowledge. The reliability of the scale, as evaluated by Ondruseket al [10].

The relationship between independent variables and mean breast cancer knowledge and main anxiety scores were evaluated by using Independent Samples t-Test and one-way ANOVA. A.

Fortunately, the missing data were few; it was dealt with them by distributing them evenly overall data and comparing the results before and after adding them. There was no difference in results.

### 2.4 Ethical consideration

Informed consent was obtained before filling out the questionnaires.

## 3 Results

This study assessed the level of awareness among medical female students in Syrian Private University.

The total participants were 407 divided into three faculties (medicine, dentistry, pharmacy) (See Table 1).

**Table 1:** Numbers of participants in each college (n = 407).

	Total No.	Due to years					
		1^st^	2^th^	3^th^	4^th^	5^th^	6^th^
Medicine	205	39	47	46	31	29	13
Dentistry	78	16	30	17	10	5	
Pharmacy	124	14	22	27	33	28	
Total	407	69	99	90	74	62	13

### 3.1 Breast cancer knowledge

There are four axes; each of them shows the side of breast cancer awareness.

The average knowledge rate was (57.5%). This rate varied between sections (See Table 2).

**Table 2:** Knowledge Percentage in Each Axis (n = 407).

	Axis 1	Axis 2	Axis 3	Axis 4
No.	407	407	407	407
(%)	65.90	56.75	57.47	61.79

(71 %) of the total participants said they are familiar with early detection methods of breast cancer and (70 %) of participants were not familiar with mammography. The students who were examined by mammography were about (37%). It was also clear that most of the future-doctors participants believe that tight bra can cause breast cancer, (72 %) answered yes, which is a huge rate.

### 3.2 Breast cancer symptoms and signs

The overall knowledge among the participants were (65.7%) symptoms which is not compatible with the expected academic level of students in medical faculties. Approximately (59%) didn't know that the rash is a symptom of breast cancer and (74 %) thought that axillary pain is a complaint. It was very obvious that the majority of students believed that painful lump can be cancerous, (81%) admitted that. (60 %) of females did not know that the increase of breast mass can indicate breast cancer.

### 3.3 Breast cancer risk factors

The most common risk factors according to the students was the hormone replacement therapy (HRT) after menopause, the rate reached (72.5%), among The students, (66%) did not know that precocious puberty can lead to cancer and (51 %) did not believe that delayed menopause is a risk factor. Other variables such as gender, academic, marital status, having doctor relative and having an affected relative did not have a significant influence on the level of understanding of risk factors (for p-values see Table 3). It was stupendous that the students in pharmacy understand risk factor (60%) more than participants from medicine (57%). (51%) of the students did not consider overweight as a risk factor.

**Table 3:** Demographic Characteristics of Study Sample (n = 407).

Characteristics	No.	%
Age		

17–19 year	141	34.6
20–22 year	216	53.1
23–25 year	39	9.6
more than 25 year	11	2.7

Marital status		

Single	395	97.1
Married	12	2.9

Economic status		

Bad	3	0.7
Moderate	97	23.8
Good	182	44.7
Excellent	125	30.7

Family history of breast cancer		

Yes	76	18.7
No	331	81.3

Medical relative		

Yes	298	73.2
No	109	26.8

Religion		

Muslim	384	94.3
Christian	23	5.7

### 3.4 Breast self-examination (BSE) practice

It is a good result that (86.7 %) are familiar with self-examination and (94.8%) believe it is very important. However, it was obvious that the practical side was poor, there were (32.7%) of students who apply BSE. (84.5%) agreed on the necessity of BSE even without any symptoms.

**Figure 1:**
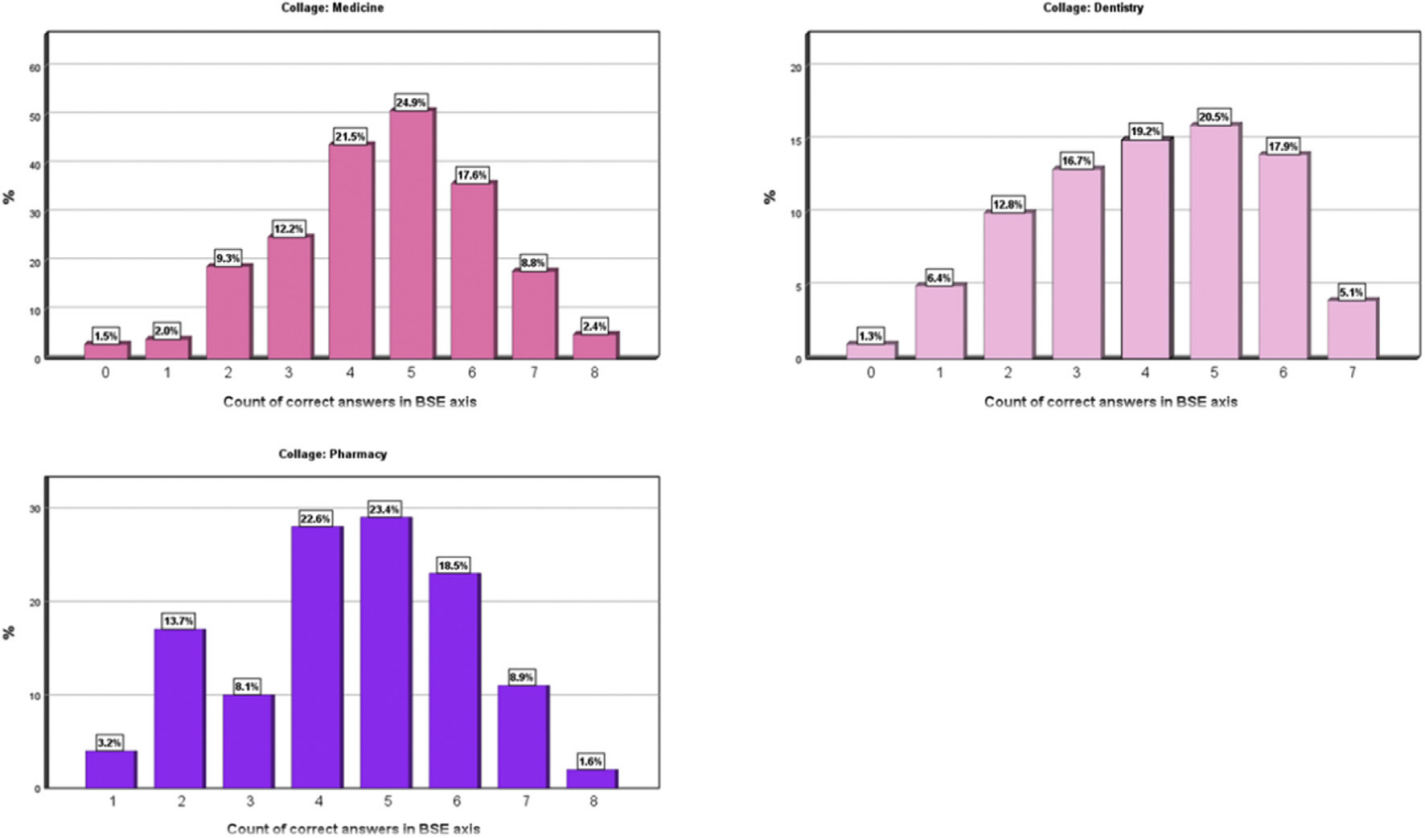
Show charts that clarify numbers of correct answers in BSE axis according to collage.

The charts in figures and tables sections [Figure 1]) show that female medical students had responded to a larger number of correct answers compared to the College of Pharmacy and the College of Dentistry students.

In the sample of female students who did not have a self-examination before, we found statistical differences (p-value = 0.012 < 0.05) among those who are afraid and those who are not afraid of breast cancer as a concern, where the difference was in favour to the group of students who are afraid of breast cancer (72.3% (196)vs. 27.7% (75)). So, the fear of breast cancer was a barrier of BSE among students.

However, in the sample of female students who believed that breast screening was painful, there was a statistical difference (p-value = 0.003 < 0.05) between those who did not practice self-examination before. This difference was in favour of the sample of those who did not perform self-examination (68.6 % (48) versus 31.4 % (22)). We see that fear of painful examination prevents young females from performing BSE.

### 3.5 Correlations with knowledge percentage and questions of the survey

There were statistically significant differences (p-value = 0.00 < 0.05) in the percentage of average knowledge and in each axis of the survey among those who had completed curriculum that included breast cancer in the Faculty of Medicine, such as pathology - general surgery – gynaecology, where the percentage of knowledge increases with the passing courses.

There were statistically significant differences (p-value = 0.036 < 0.05) in the percentage of knowledge in the first axis (general information axis) among persons who had a first-degree relative and who had not to have a first-degree relative. The difference was in favour of those who have a first-degree relative (72% VS 65.09%).

In our country, the years of studying medicine are six. When comparing the students’ knowledge with thestudying years, were found statistically and significantly different compared to total knowledge (p-value = 0.00 < 0.05) and knowledge in each axis for the different study years. The percentage of knowledge increases from the first to the fifth year, decreasing slightly in the sixth year except in the fourth axis (BSE axis). (See Table 4 for p-values).

**Table 4:** Knowledge Percentage of each axis comparing with study year in Medicine Collage (n = 407).

		N	Mean	Sig.
Axis 1 (General Information)	1	39	62.14	0.008
	2	46	66.75	
	3	46	67.77	
	4	30	70.19	
	5	28	73.31	
	6	11	72.19	
	Total	200	67.82	
Axis 2 (Signs & Symptoms)	1	39	51.67	0.00
	2	46	52.67	
	3	46	61.70	
	4	30	66.66	
	5	28	75.27	
	6	11	73.42	
	Total	200	60.96	
Axis 3 (Risk Factors)	1	39	48.71	0.00
	2	46	55.73	
	3	46	57.31	
	4	30	57.87	
	5	28	70.77	
	6	11	70.24	
	Total	200	57.95	
Axis 4 (BSE∗)	1	39	55.12	0.00
	2	46	58.33	
	3	46	62.86	
	4	30	68.88	
	5	28	72.02	
	6	11	77.27	
	Total	200	63.29	
Average of Knowledge Percentage	1	39	54.41	0.00
	2	46	58.37	
	3	46	62.41	
	4	30	65.90	
	5	28	72.84	
	6	11	73.28	
	Total	200	62.50	

∗ Breast Self-Examination.

Meanwhile, the pharmaceutical studies include five years, there were statistically significant differences (p-value = 0.004 < 0.05) in total knowledge due to years, which increases with the year from the first to the fourth year, decreasing in the fifth year.

There are statistically significant differences (p-value = 0.001 < 0.05) in the knowledge of the first axis (the general information axis) in different years, which increases from the first to the fourth year to decrease in the fifth.

There are statistically significant differences (p-value = 0.006 < 0.05) in the third axis (risk factors) for the different academic years where the percentage of knowledge increases with the year from the first to the fifth year. In other sections of the survey, there are not any statistically significant differences. (see Table 5).

**Table 5:** Knowledge Percentage of each axis comparing with study year Pharmacy Collage (n = 407).

		N	Mean	Sig.
Axis 1 (General Information)	1	14	53.78	0.001
	2	22	67.00	
	3	27	65.35	
	4	33	69.34	
	5	28	68.90	
	Total	124	66.22	
Axis 2 (Signs & Symptoms)	1	14	49.45	0.831
	2	22	55.24	
	3	27	55.84	
	4	33	55.94	
	5	28	54.94	
	Total	124	54.83	
Axis 3 (Risk Factors)	1	14	46.75	0.006
	2	22	52.89	
	3	27	58.92	
	4	33	63.91	
	5	28	68.83	
	Total	124	60.04	
Axis 4 (BSE∗)	1	14	52.97	0.127
	2	22	64.77	
	3	27	62.34	
	4	33	66.91	
	5	28	62.20	
	Total	124	62.90	
Average of Knowledge Percentage	1	14	50.74	0.004
	2	22	60.00	
	3	27	60.61	
	4	33	64.02	
	5	28	63.72	
	Total	124	61.00	

∗ Breast Self-Examination.

There were no statistically significant differences in knowledge between years in Dentistry Collage.

It was a good result that (72%) knew what the specialty to be reviewed was. That related with older age with statistically significant differences (confidence interval = 95%, -value = 0.018 < 0.05).

In the sample of female students who believed that mammography is painful, there were statistical differences between those who perform the examination and those who did not. This difference was for the sample of those who did not perform the mammogram (73.5% (50) vs. 26.5% (18), that means the majority believed it causes pain. (P-value = 0.008 < 0.05).

### 3.6 Attitudes and feelings towards breast cancer

The idea of breast cancer was terrifying for (69.8%) of females. (56%) feel embarrassed if someone checks their breasts and the majority of female students (82%) preferred to be examined by a female doctor. (56%) of participants are fearful of being examined by rude doctors.

### 3.7 Comparison with other studies

Overall, our study is in agreement with previous studies have done in other parts of the world and it showed the general lack of adequate knowledge on breast cancer by universities students in Syria. A study in Angola showed most of the participants (97.5% of medical students and 98.5% of non-medical students) indicated the need for more information on breast cancer to be provided in high school and university; thus, suggesting a willingness to learn more about the disease. In Tunisia, studies revealed poor knowledge of breast cancer and the screening methods as well as low levels of practice in breast cancer screening among women [5].

## 4 Discussion

Population-based cancer screening is a much more complex public health undertaking than early diagnosis and is usually cost-effective when done in the context of high-standard programs that target all the population at risk in a given geographical area with high specific cancer burden, with everyone who takes part being offered the same level of screening, diagnosis and treatment services. Also, big role falls on doctors, nurses and medical students.

The main goal of this study was to assess breast cancer awareness and knowledge among university students.

The present study showed that (71%) of students said they know about early detection methods of breast cancer while (70%) of them did not know mammography, such confusion, shows that medical students know about tools of diagnosis just theoretically, similar results in a study in Saudi Arabia that presented (78%) do not know mammography [11].

(72%) of the sample said that tight bra can cause breast cancer. In medical literature, no aspect of wearing a bra, including cup size, the average number of hours/day worn, wearing a bra with an underwire, or the first time she began regularly wearing a bra, was associated with risks of either intraductal carcinoma) IDC or (intralobular carcinoma) ILC. Results of another study did not support an association between bra wearing and increased breast cancer risk among postmenopausal women [12].

The present review demonstrates insufficient evidence to establish a positive association between the duration and type of brassiere wearing and breast cancer [13]. Therefore, this point must be clear in the mind of medical students because it is a common question among women in every community.

A majority (81%) of participants in this study considered that pain is an important symptom of breast cancer. This, as reported by Powe et al [14], is a widespread misconception as most people associate pain with the occurrence of cancer. In fact, pain is not necessarily an early symptom of breast cancer. Our result was agreed with a study among university students in Angola by Sambanje et al [15] that shows 80 % of students thought that lumps in the breast which are cancerous would be painful. Perhaps that is a big problem for us is that the sample quality included future doctors.

While the average knowledge of risk factors is (57 %), it is still considered a low rate among medical students, but that is similar to many studies in Angola [15], Saudi Arabia [11].

The students (66%) did not know the precocious puberty can lead to cancer and (51%) did not believe the delayed menopause is a risk factor. Overweight was not identified as a risk factor by (51%) of participants. This was reported; too by Sambanje et al [15] that (57%) did not consider being overweight could cause breast cancer.

(72%) of females said that oral conceptive is a risk factor while in Angola [15]more than 60% of students were not aware of the risk associated with the use of oral contraceptive and hormone replacement therapy (HRT).

Unfortunately, students’ understanding of symptoms and risk factors is inadequate that may delay diagnosis and therefor treatment. As reported by Ukwenya AY et al in Nigeria, the study showed that delayed treatment of symptomatic breast cancer is a result of lack of awareness [16].

Interestingly, even though a majority of the participants (95 %) appreciated the need for BSE, it was evident that (49%) students had not received proper information on how to properly perform it, which goes hand in hand with results in Angola study [15].

(86 %) of students had heard about BSE, but 51% only knew how to practice that there is a big argue between studies about BSE. In META-ANALYSIS reported in 2003 ′Women should, of course, still be aware of changes in their breasts and seek advice if concerned, but being taught BSE and practising it regularly is no more effective at reducing breast cancer mortality than finding the tumour by chance'' [17].

In 2001, a literature review found that BSE is associated with considerably more women seeking medical advice and having biopsies, but is not an effective method of reducing breast cancer mortality [18].

Old studies reported that tumours found during BSE and routine examination of the breast averaged 6.1 mm smaller in diameter than those discovered accidentally [19].

Although confusion and lack of studies that confirm the role of BSE in reducing mortality, the importance of early diagnosis cannot be ignored. It was found that breast inspection and careful palpation during BSE associated with less extensive disease, this finding suggests that properly executed breast examination may lead to early diagnosis [20].

There were (32.7%) of students who participate BSE that agree with study in Korea where (27%) performed BSE that justifies the lack of knowledge about BSE [10].

One of the barriers that may prevent females from doing BSE, that (68%) believed it was painful. Therefore, we must teach the students how to practice BSE and confirm that it does not cause any pain [21].

There is another way for an early diagnosis like mammography; it is, now, the most effective method for the early detection of breast cancer. In most published studies, it had been demonstrated that screening mammography could significantly reduce mortality attributable to breast cancer among women older than 50 years of age [22, 23, 24].

There were (71%) said that they knew early detection methods of breast cancer. This rate is not real because (70 %) of the participant did not hear about mammography, and (68%) did not perform BSE although (86 %) heard about it. It is very disappointing that doctors of the future don't have practical experience about early screening way of breast cancer.

## 5 Conclusion

Although our sample is the students of the medical colleges who should be more informed than others about early detection methods of breast cancer, but the results were the opposite of the expected, so it is necessary to search for the reasons whether it is in the curriculum itself, the teaching ways. or that the students do not link what they learn in colleges in practical life. Perhaps the most important reason is the lack of awareness programs that must include all strata of society, especially students of medical colleges and doctors for their important role in spreading awareness to avoid this danger that surrounds our ladies.

## Declarations

### Author contribution statement

A. Omar and A. Bakr: Conceived and designed the experiments; Performed the experiments; Analyzed and interpreted the data; Contributed reagents, materials, analysis tools or data; Wrote the paper.

N. Ibrahim: Conceived and designed the experiments; Analyzed and interpreted the data; Contributed reagents, materials, analysis tools or data.

### Funding statement

This research did not receive any specific grant from funding agencies in the public, commercial, or not-for-profit sectors.

### Competing interest statement

The authors declare no conflict of interest.

### Additional information

No additional information is available for this paper.
